# Gut microbiota deficiency reduces neutrophil activation and is protective after ischemic stroke

**DOI:** 10.1186/s12974-025-03448-w

**Published:** 2025-05-23

**Authors:** Ali A. Tuz, Susmita Ghosh, Laura Karsch, Medina Antler, Vivian Lakovic, Sabrina Lohmann, Amber Hope Lehmann, Alexander Beer, Dennis Nagel, Marcel Jung, Nils Hörenbaum, Viola Kaygusuz, Altea Qefalia, Belal Alshaar, Niloufar Amookazemi, Silvia Bolsega, Marijana Basic, Jens T. Siveke, Sven Heiles, Anika Grüneboom, Smiths Lueong, Josephine Herz, Albert Sickmann, Nina Hagemann, Anja Hasenberg, Dirk M. Hermann, Matthias Gunzer, Vikramjeet Singh

**Affiliations:** 1 Institute for Experimental Immunology and Imaging, University Hospital Essen, University of Duisburg-Essen, 45147 Essen, Germany; 2 Leibniz-Institut Für Analytische Wissenschaften - ISAS - E.V., 44139 Dortmund, Germany; 3 Institute for Laboratory Animal Science and Central Animal Facility, Hannover Medical School, 30625 Hannover, Lower-Saxony Germany; 4 Division of Solid Tumor Translational Oncology, German Cancer Consortium (DKTK, Partner Site Essen), German Cancer Research Center (DKFZ), Heidelberg, Germany; 5 Lipidomics, Faculty of Chemistry, University of Duisburg-Essen, 45141 Essen, Germany; 6 Department of Pediatrics I, Neonatology and Experimental Perinatal Neurosciences, and Center for Translational Neuro- and Behavioral Sciences (C-TNBS), University Hospital Essen, University of Duisburg-Essen, Essen, Germany; 7 Medical Faculty of the Ruhr-University Bochum, Universitätsstraße 150, Bochum, 44801 Germany; 8 Department of Neurology, University Hospital Essen, University of Duisburg-Essen, 45147 Essen, Germany

**Keywords:** Gut microbiota, Neutrophils, Inflammation, Ischemic stroke

## Abstract

Neutrophils are readily activated immune cells after ischemic stroke in mice and patients. Still, the impact of gut microbiota on neutrophil activation and its influence on inflammatory brain injury remain undefined. We report that natural microbiota colonization of germ-free (GF) mice induces substantial neutrophil activation and deteriorates stroke pathology. The colonized Ex-GF stroke mice had considerably larger infarct sizes and higher sensorimotor deficits than GF littermates. Furthermore, employing an antibiotic-based mouse model of microbiota deficiency, we demonstrate that gut microbiota depletion induces a juvenile neutrophil phenotype characterized by the upregulation of resting state surface receptors, reduced inflammatory proteins, and levels of circulating NETs. This disarming of neutrophil responses was associated with decreased expression of brain inflammatory genes, vascular thrombus formation, reduced infarct size, and alleviated behavioral deficits. We conclude that gut microbes strongly influence neutrophil activation after stroke and thus directly contribute to stroke severity.

**Supplementary Information:**

The online version contains supplementary material available at 10.1186/s12974-025-03448-w.

## Highlights

• Cohousing transfers gut microbiota to germ-free mice, increasing neutrophil activation.

• Antibiotics-induced microbiota deficiency reduces neutrophil activation and protects the stroke brain.

• Microbiota deficiency reduces circulating NETs, brain vascular thrombus formation and neuroinflammation after stroke.

**Supplementary Information:**

The online version contains supplementary material available at 10.1186/s12974-025-03448-w.

## In brief

Tuz et al. demonstrate the role of gut microbiota in driving neutrophil activation and deteriorating stroke outcome. Antibiotic-mediated depletion of gut microbiota reduced neutrophil inflammatory proteins, neutrophil extracellular traps (NETs) release, and ensuing brain inflammation.

## Introduction

Clinical studies have reported an association between elevated circulating neutrophil numbers and long-term adverse stroke outcome [1]. This is further supported by experimental data showing that the infiltration of activated neutrophils into the ischemic brain exacerbates tissue injury [2, 3]. However, such studies have not comprehensively identified systemic signals that trigger neutrophil activation. The modulation of the progression of different neurological disorders by gut microbiota transplantation implies an effect of microbes on systemic immunity [4, 5]. Our previous studies demonstrated that the transplantation of dysbiotic microbiota into germ-free mice resulted in stroke progression [4]. In addition, stroke-induced intestinal barrier permeability can promote the translocation of intestinal microbes to systemic tissues, which may increase inflammation [6]. However, despite the striking effects of neutrophils and gut microbiota on stroke pathology, studies investigating the impact of their interactions on disease progression have not yet been reported.

Activated neutrophils can induce neuronal tissue injury through multiple mechanisms, including the secretion of pro-inflammatory cytokines and neutrophil extracellular traps (NETs) [2]. NETs with activated platelets contribute to microvascular thrombus formation in the ischemic brain [7]. We have previously demonstrated that NET-induced microvascular thrombus formation in intestinal lymphoid tissue leads to cellular apoptosis and immunodeficiency after stroke [8]. In addition, metabolic disturbances that are often present in patients with cardiovascular disease can augment NET release and worsen disease outcome [9]. Recent reports have revealed that commensal microbes can induce neutrophil activation phenotypes [10]. Reciprocally, the depletion of gut microbiota may lead to the disarmament of neutrophil neurotoxic functions and stimulate tissue protective phenotypes.

There is strong evidence that gut microbiota can impact immune responses and infarct development after ischemic stroke [11, 12]. Interestingly, gut microbiota depletion with antibiotics was demonstrated to reduce brain infarcts in a mouse model of transient stroke [13]. Notably, the early brain infiltration of systemically activated neutrophils is detrimental to the ischemic brain [14]. Because of the critical involvement of neutrophils and gut microbiota in stroke pathology, we sought to delineate the causal relationship between them using two different mouse models. Here, we used germ-free and microbiota-depleted stroke mice to investigate whether gut microbiota influence neutrophil activation and affect disease severity.

## Results

### Commensal microbes drive neutrophil activation that worsens stroke progression

**Fig. 1:**
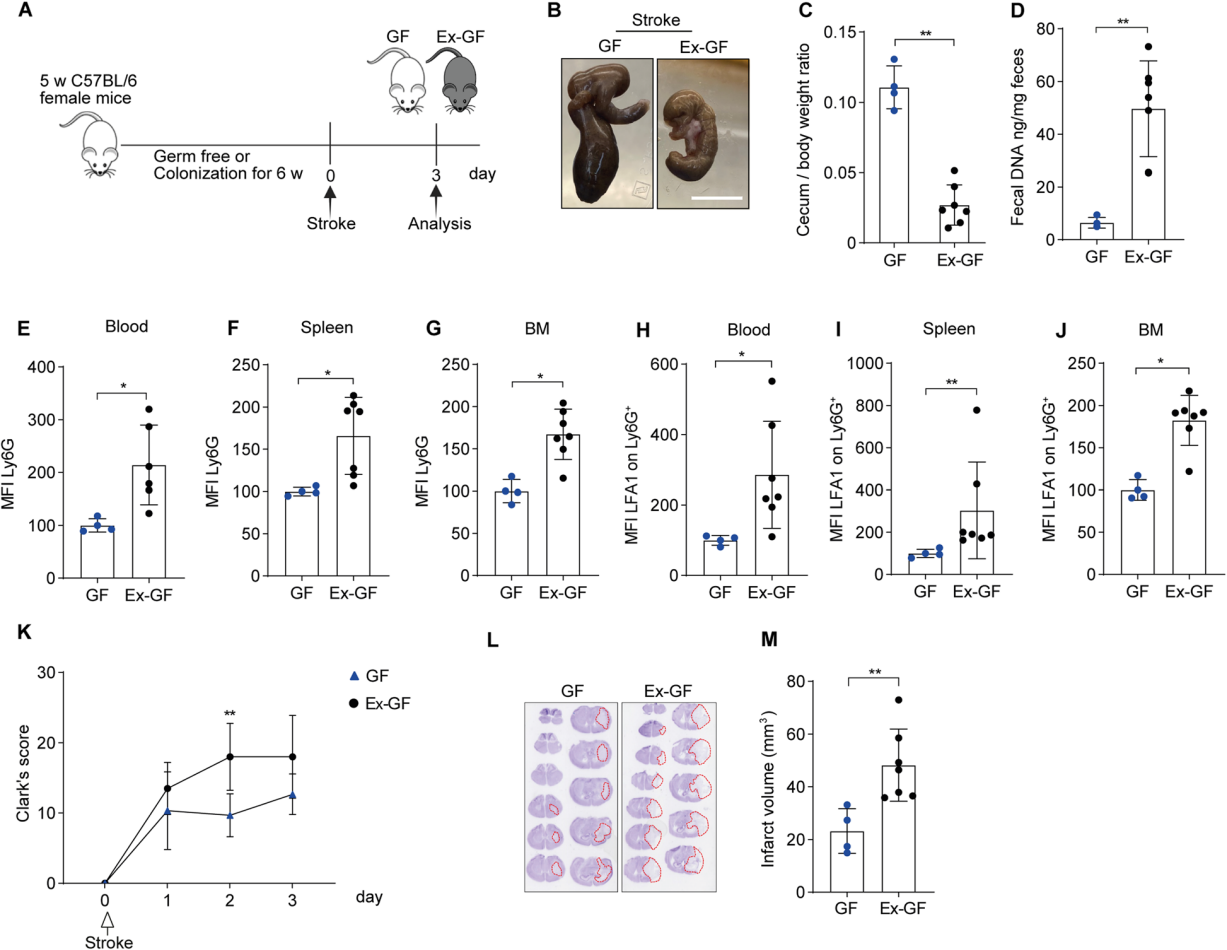
Gut microbiota trigger neutrophil activation, and increase brain infarcts and sensorimotor deficits. **A** Scheme illustrating the experimental design. **B** Images showing caecum size in GF and Ex-GF stroke mice. Scale bar = 1 cm. **C** Cecum/body weight ratio in GF and Ex-GF stroke mice. **D** Total bacterial DNA (ng) per mg fecal samples from GF and Ex-GF stroke mice. **E–G** Mean fluorescence intensity (MFI) of Ly6G on neutrophils in blood, spleen and tibial BM in GF and Ex-GF stroke mice three days after surgery. Values are normalized to GF controls and presented as percentages relative to the 100% MiS mean. **H-J** MFI of LFA1 on neutrophils in blood, spleen, and tibial BM in GF and Ex-GF stroke mice. **K** Clark’s score for GF and Ex-GF mice after one to three days of ischemic stroke. **L** Representative images of cresyl violet stained brain sections from GF and Ex-GF stroke mice. The red outline marks the infarct regions. **M** Brain infarct volumes (mm^3^) of GF and Ex-GF mice after three days of stroke. Data are analyzed by the unpaired Mann–Whitney U test, *p < 0.05, **p < 0.01, *n* = 4–7 mice per group. GF = germ-free, BM = bone marrow

First, we investigated the effects of microbial colonization in germ-free (GF) mice on neutrophil activation after transient ischemic stroke (Fig. 1A). Co-housing of GF mice with SPF mice for 6 weeks resulted in an increase in total fecal DNA and a reduced caecum-body weight ratio (Fig. 1B-D), indicating effective gut microbial colonization [15].

Furthermore, we analyzed the expression of neutrophil activation markers in GF and Ex-GF mice using multicolor flow cytometry (Figure S1 A). We found that microbiota colonization in mice leads to the increased surface expression of Ly6G on neutrophils in the blood, spleen and tibial bone marrow (BM) after stroke (Fig. 1E-G). In addition, there was an increased level of leukocyte function antigen (LFA)−1 on neutrophils in blood, spleen, and BM of Ex-GF mice compared to GF stroke mice (Fig. 1H-J). Microbiota-colonized Ex-GF stroke mice showed a slight reduction in the percentage of CD62L^+^ immature neutrophils [16] in the blood (< 1%) and spleen but not in BM (Figure S1B-D) and a higher frequency of CXCR4^+^ activated neutrophils [17] in the blood and spleen, but not in BM (Figure S1E-G). Notably, Ex-GF mice showed higher brain infarcts and behavioral deficits (Tables 1 and 2) after stroke than their GF littermates (Fig. 1K-M) without significantly affecting neutrophil numbers in blood, spleen, and BM (Figure S1H-J). These data suggest that gut microbiota colonization may induce the maturation of systemic neutrophils and promote tissue injury after stroke.

**Table 1:** General Neuroscore

Time-point of scoring		***score***
Hair	0. Hair neat and clean	
	1. Localized piloerection and dirty hair in 2 body parts (nose and eyes)	
	2. Piloerection and dirty hair in > 2body parts	
Ears (mouse on an open bench top)	0. Normal (ears are stretched laterally and behind, they react by straightening up following noise)	
	1. Stretched laterally but not behind (one or both), they react to noise	
	2. Same as 1. NO Reaction to noise	
Eyes (mouse on OBT)	0. Open, clean and quickly follow the surrounding environment	
	1. Open and characterized by aqueous mucus. Slowly follow the surrounding environment	
	2. Open and characterized by dark mucus	
	3. Ellipsoidal shaped and characterized by dark mucus	
	4. Closed	
Posture (place the mouse on the palm and swing gently)	0. The mouse stands in the upright position with the back parallel to the palm. During swing, it stands rapidly	
	1. The mouse stands humpbacked. During the swing, it flattens the body to gain stability	
	2. The head or part of the trunk lies on the palm	
	3. The mouse lies on one side, barely able to recover the upright position	
	4. The mouse lies in a prone position, not able to recover the upright position	
Total score for general scoring (normal = 0 max = 12)

**Table 2:** Focal Neuroscore

Time-point of scoring		***score***
Body symmetry (mouse on OBT, observe the nose-tail line)	0. Normal (Body: normal posture, trunk elevated from the bench, with fore and hindlimbs leaning beneath the body. Tail: straight)	
	1. Slight asymmetry (Body: leans on one side with fore and hindlimbs leaning beneath the body. Tail: slightly bent.)	
	2. Moderate asymmetry (Body: leans on one side with fore and hindlimbs stretched out. Tail: slightly bent)	
	3. Prominent asymmetry (Body: bent, on one side lies on the OBT. Tail: bent)	
	4. Extreme asymmetry (Body: highly bent, on one side constantly lies on the OBT. Tail: highly bent)	
Gait (mouse on OBT. Observed undisturbed)	0. Normal (gait is flexible, symmetric and quick)	
	1. Stiff, inflexible (humpbacked walk, slower than normal mouse)	
	2. Limping, with asymmetric movements	
	3. Trembling, drifting, falling	
	4. Does not walk spontaneously (when stimulated by gently pushing the mouse walks no longer than 3 steps)	
Climbing (mouse on a 45° surface. Place the mouse in the center of the gripping surface)	0. Normal (mouse climbs quickly)	
	1. Climbs with strain, limb weakness present	
	2. Holds onto slope, does not slip or climb	
	3. Slides down slope, unsuccessful effort to prevent fail	
	4. Slides immediately, no effort to prevent fail	
Circling behavior (mouse on OBT, free observation)	0. Absent circling behavior	
	1. Predominantly one-side turns	
	2. Circles to one side, although not constantly	
	3. Circles constantly to one side	
	4. Pivoting, swaying, or no movement	
Forelimb symmetry (mouse suspended by tail)	0. Normal	
	1. Light asymmetry: mild flexion of contralateral forelimb	
	2. Marked asymmetry: marked flexion of contralateral limb, the body slightly bends on the ipsilateral side	
	3. Prominent asymmetry: contralateral forelimb adheres to the trunk	
	4. Slight asymmetry, no body/limb movement	
Compulsory circling (forelimbs on bench, hindlimbs suspended by the tail: it reveals the presence of the contralateral limb palsy)	0. Absent. Normal extension of both forelimbs	
	1. Tendency to turn to one side (the mouse extends both forelimbs, but starts to turn preferably to one side)	
	2. Circles to one side (the mouse turns towards one side with a slower movement compared to healthy mice)	
	3. Pivots to one side sluggishly (the mouse turns towards one side failing to perform a complete circle)	
	4. Does not advance (the front part of the trunk lies on the bench, slow and brief movements)	
Whisker response (mouse on the OBT)	0. Normal	
	1. Light asymmetry (the mouse withdraws slowly when stimulated on the contralateral side)	
	2. Prominent asymmetry (no response when stimulated to the contralateral side)	
	3. Absent response contralaterally, slow response when stimulated ipsilaterally	
	4. Absent response bilaterally	
Total score for focal deficits (normal = 0 max = 28)		

### Disarming neutrophil activation by gut microbiota depletion improves stroke recovery

**Fig. 2:**
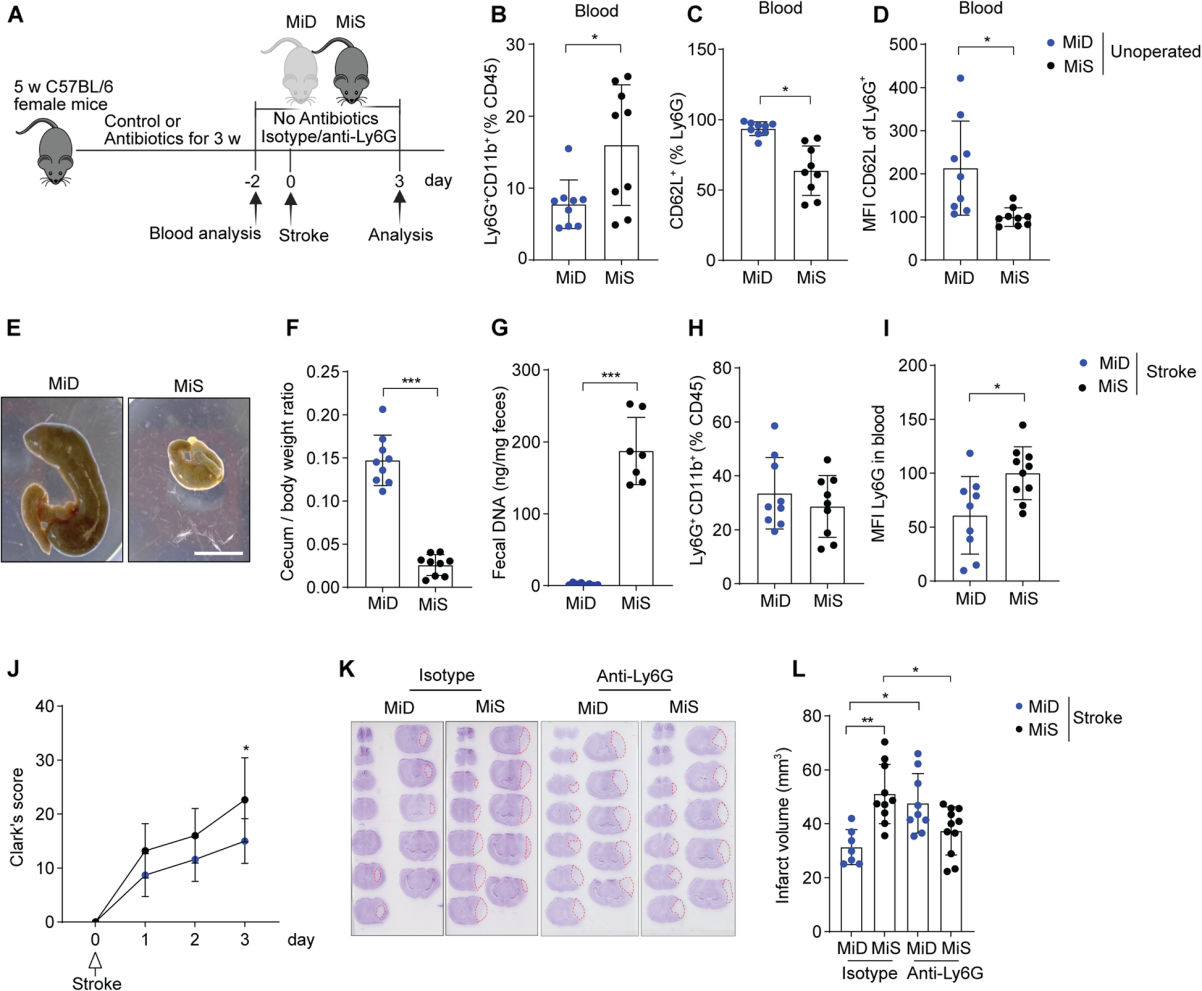
Gut microbiota deficiency reduces neutrophil activation and is protective to injured stroke brain. **A** Scheme illustrating experimental design. **B** The percentages of Ly6G^+^CD11b^+^ blood neutrophils in unoperated MiD and MiS mice. **C** The frequency of CD62L^+^ neutrophils in the blood of MiD and MiS unoperated mice. **D** Mean fluorescence intensity (MFI) of CD62L^+^ on blood neutrophils in MiD and MiS unoperated mice. Values are normalized to MiS controls and presented as percentages relative to the 100% MiS mean. **E** Images showing caecum size in MiD and MiS stroke mice three days after surgery. Scale bar = 1 cm. **F** Cecum-body weight ratio in MiS and MiD stroke mice. **G** Total bacterial DNA (ng) per mg fecal samples from MiD and MiS stroke mice. **H** The frequency of Ly6G^+^CD11b^+^ blood neutrophils in MiD and MiS stroke mice three days after surgery. **I** MFI of Ly6G in blood neutrophils of MiD and MiS stroke mice. Values are normalized to MiS controls and presented as a percent decrease. **J** Clark’s score for MiD and MiS mice after one to three days of ischemic stroke. **K** Representative images of cresyl violet stained brain sections of MiD and MiS stroke mice treated with isotype or anti-Ly6G antibody. The red outline marks the infarct regions. **L** Brain infarct volumes of MiD and MiS mice after three days of stroke. Data are analyzed by the unpaired Mann–Whitney U test for two groups and the Kruskal–Wallis test for multiple comparisons, *p < 0.05, **p < 0.01, ***p < 0.001, *n* = 7–10 mice per group. MiD = microbiota-deficient, MiS = microbiota sufficient

Early life interactions between gut microbiota and neutrophils can generate innate immune memory and protect the host from infections [18, 19]. Hence, beyond using GF mice with overall microbiota deficiency since birth, we investigated whether microbiota depletion in conventionally reared SPF mice could affect neutrophil activation and stroke severity (Fig. 2A). Microbiota depletion using a mixture of antibiotics for three weeks resulted in a reduced frequency of circulating neutrophils in unoperated microbiota-deficient (MiD) mice compared to microbiota-sufficient mice (MiS) (Fig. 2B). We identified higher frequencies of CD62L^+^ immature neutrophils and increased expression of CD62L on circulating neutrophils in MiD compared to MiS mice (Fig. 2C, D). Expression of neutrophil maturation receptors CXCR2 and CXCR4 in blood was reduced in MiD compared to MiS unoperated mice (Figure S2B-C). These results suggest that the maturation of neutrophils was reduced in mice with a deficiency of gut microbiota.

Furthermore, both groups of mice underwent tMCAO and were analyzed three days after surgery. Microbiota depletion increased cecum size, cecum/body weight ratio, and decreased fecal DNA content in MiD compared to MiS mice (Fig. 2E, F, G). We found a similar frequency of circulating neutrophils in MiS and MiD stroke with a significant reduction in the neutrophil maturation receptor Ly6G expression in MiD mice (Fig. 2H, I). Of note, the number of Ly6G^+^CD11b^+^ neutrophils in the blood, spleen, and tibial bone marrow remained unchanged in MiD and MiS stroke mice (Figure S2D-F).

Interestingly, the depletion of gut microbiota in MiD mice significantly reduced sensorimotor deficits (Tables 1 and 2) and cerebral infarct volume compared to MiS mice three days after stroke (Fig. 2J, K, L). To reveal if the reduced brain injury in MiD mice also corroborated with lower inflammatory signaling, we purified total RNA from the ischemic hemispheres of these MiD and MiS mice and analyzed transcriptomic changes by bulk RNA sequencing and bioinformatic analysis. Our results showed that microbiota depletion triggered very low levels of several inflammatory signaling genes such as Myd88 [20], IL-17ra [21], Edn1 [22] and Stat3 [23] in the ischemic hemispheres of MiD mice compared to MiS stroke mice (Figure S2G). Notably, the pathways responsible for inflammatory signaling, such as TNF-α [24], Stat5, IL-6, and Jak-stat [25] were decreased in MiD mice compared to MiS stroke mice, suggesting reduced brain inflammation in deficiency of gut microbiota (Figure S2H).

Expecting a critical contribution of activated neutrophils in infarct development, their antibody-mediated depletion in MiS mice reduced cerebral infarcts compared to neutrophil-sufficient MiS mice (Fig. 2L). In contrast, neutrophil depletion in MiD mice increased brain infarcts compared to neutrophil-sufficient MiD mice, suggesting a plausible protective function of immature neutrophils. Together, these data indicate that gut microbiota-dependent neutrophil activation may contribute to the promotion of cerebral injury after stroke. Most importantly, immature neutrophils in microbiota-deficient mice appear to have cerebral tissue-protective functions.

Next, we investigated the effect of gut microbiota-neutrophil interactions on microglia responses in MiS and MiD stroke mice. Microglia were analyzed three days after stroke in the infarct core and lesion border in the ipsilateral hemispheres (Figure S3 A). The total number of Iba1^+^ microglia in the infarct core or the border regions did not differ between MiD and MiS stroke mice (Figure S3B, C). Using an automated morphological analysis of Iba1^+^ cells in the perilesional areas [26] (Figure S3D, E), we found that microglia from neutrophil-depleted MiS stroke mice displayed significantly increased ramifications (endpoints) and process length compared to neutrophil-sufficient MiS mice, indicating a less activated microglia phenotype in the absence of neutrophils (Figure S3 F, G). In contrast, microglial morphology remained unchanged between neutrophil-sufficient MiS and MiD stroke mice. Neutrophil depletion in MiD mice resulted in a microglia activation phenotype with reduced endpoints per cell compared to MiS mice, supporting the role of microbiota-unprimed neutrophils in modulating microglia activation.

### Gut microbiota drive extensive proteomic regulation in neutrophils after stroke

**Fig. 3:**
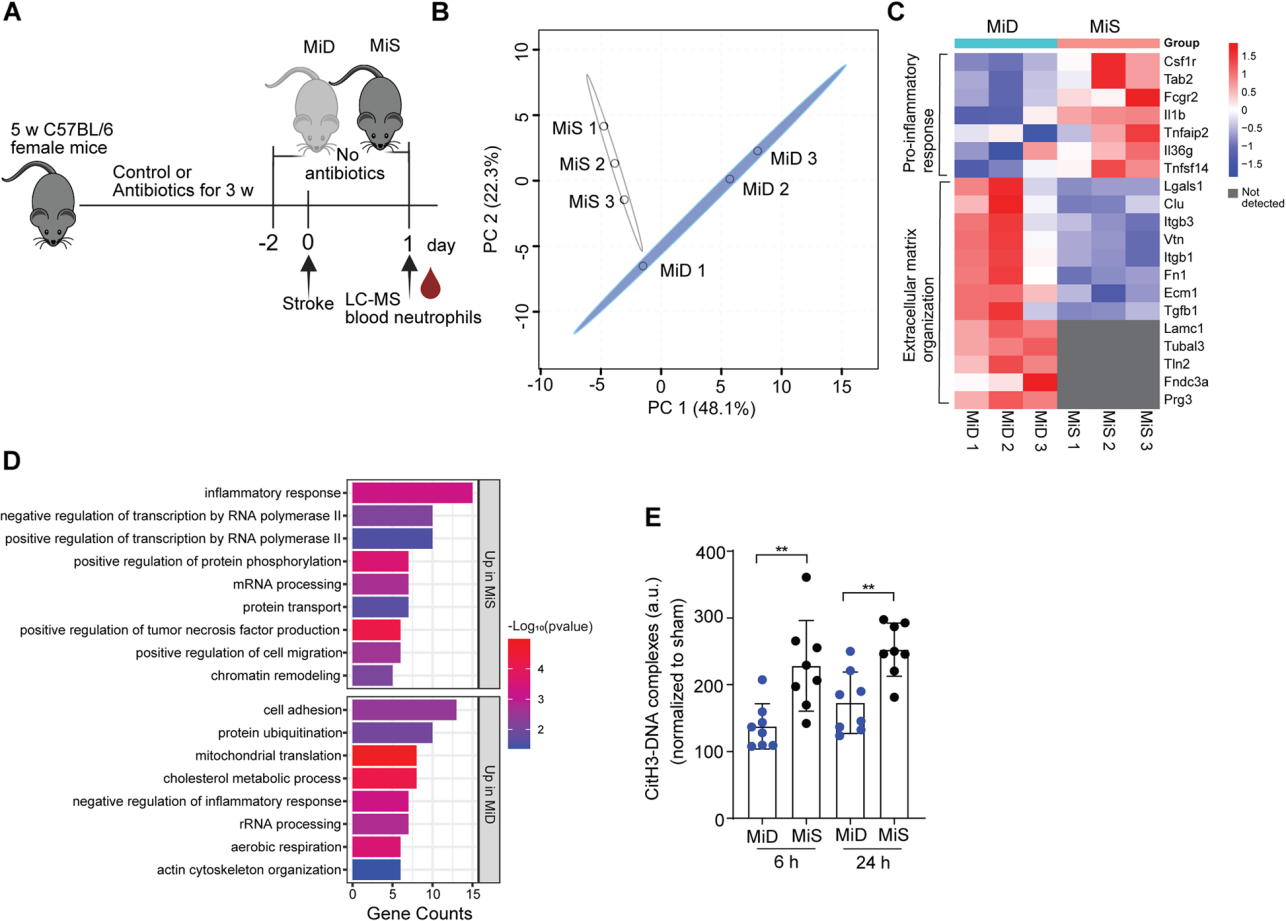
Microbiota induce inflammatory proteome in neutrophils after stroke.** A** Schematic representation of the experimental paradigm. **B** Principal component analysis (PCA) of the blood neutrophil proteome in MiD and MiS stroke mice one day after surgery (*n* = 3 mice per group). **C** Heat map illustrating differentially expressed proteins (fold change > 1.5 or < 0.5, *p*-value < 0.05) associated with proinflammatory response and extracellular matrix organization in both MiD and MiS stroke mice. The color bar represents the relative z-score, which indicates relative abundance. Red cells represent higher abundance or upregulated proteins, while blue cells represent lower abundance or downregulated proteins. **D** Functional annotation of differentially regulated proteins between two groups. The biological processes with *p*-value < 0.05 were ranked by their protein abundance, and the top eight pathways are shown here. **E** Quantification of cit-H3 complexes in plasma of MiD and MiS stroke mice at 6 h and 24 h after surgery. Data are analyzed by the unpaired Mann–Whitney U test, **p < 0.01, n = 8 mice per group. MiD = microbiota-deficient, MiS = microbiota-sufficient

Neutrophils are very early invaders to the ischemic brain and information on their molecular makeup at an earlier time after stroke is critical. Therefore, to gain a detailed insight into the microbiota-dependent neutrophil activation after stroke, we investigated the molecular composition of circulating neutrophils using liquid chromatography-based mass spectrometry (LC–MS) analysis one day after surgery (Fig. 3A).

Label-free protein quantification (LFQ) of blood neutrophils from MiD and MiS mice (*n* = 3) yielded 5,390 proteins with ≥ 1 unique peptide and 1% false discovery rate (FDR). Of these, 5,233 proteins were quantified with > 50% missing values in both groups, imputed by groupwise KNN imputation. Significant group separation was observed in supervised clustering i.e. PCA analysis based on imputed and log-transformed protein abundances (Fig. 3B). Statistical analysis revealed a total of 180 differentially regulated proteins (*p*-value < 0.05), of which 107 proteins were upregulated with fold change > 1.5 and 73 proteins were downregulated with fold change < 0.5. As expected, we found that proteins associated with pro-inflammatory responses such as Fcgr2, Csf1r, Il1b, Ccr1, and Cd300a, were upregulated in neutrophils from MiS stroke mice compared to MiD mice (Fig. 3C). These findings suggest that microbiota-neutrophil cross-talk triggers inflammatory responses after a stroke event. Conversely, we observed a significant upregulation of proteins associated with extracellular matrix organization, including extracellular matrix protein 1 (Ecm1), vitronectin (Vtn), clusterin (Clu), and fibronectin (Fn1), in circulating neutrophils from MiD stroke mice. We speculate that the increased expression of these proteins may facilitate tissue repair following neutrophil infiltration into the ischemic brain. In addition, we identified significant enrichments of proteins such as proteoglycan 3 (Prg3), talin-2 (Tln2), laminin subunit gamma-1 (Lamc1), and tubulin alpha chain-like 3 (Tubal3), which provide structural support to the extracellular matrix, exclusively in the MiD mice and not in MiS mice. Furthermore, Gene Ontology (GO) analysis of these differentially regulated proteins revealed that neutrophils from MiS mice showed upregulation in pathways related to the ‘inflammatory response,’ ‘positive regulation of tumor necrosis factor production,’ and ‘positive regulation of cell migration’ (Fig. 3D). This also corroborated with increased plasma amounts of TNF-α in MiS mice compared to MiD stroke mice (Figure S3H). In contrast, neutrophils from MiD mice showed upregulation in pathways associated with ‘actin cytoskeleton organization,’ ‘negative regulation of inflammatory response,’ and ‘mitochondrial translation.’ The reduced activation of neutrophils may also modulate their primary immune function in response to tissue injury. i.e., the release of neutrophil extracellular traps (NETs). Indeed, we found reduced levels of plasma NETs in MiD mice compared to MiS mice at 6 h and 24 h after stroke (Fig. 3E). Together, these data suggest that gut microbiota regulate the activation of systemic neutrophils, thereby promoting brain injury after stroke.

### Gut microbiota prime neutrophil activation and promote brain inflammation

**Fig. 4:**
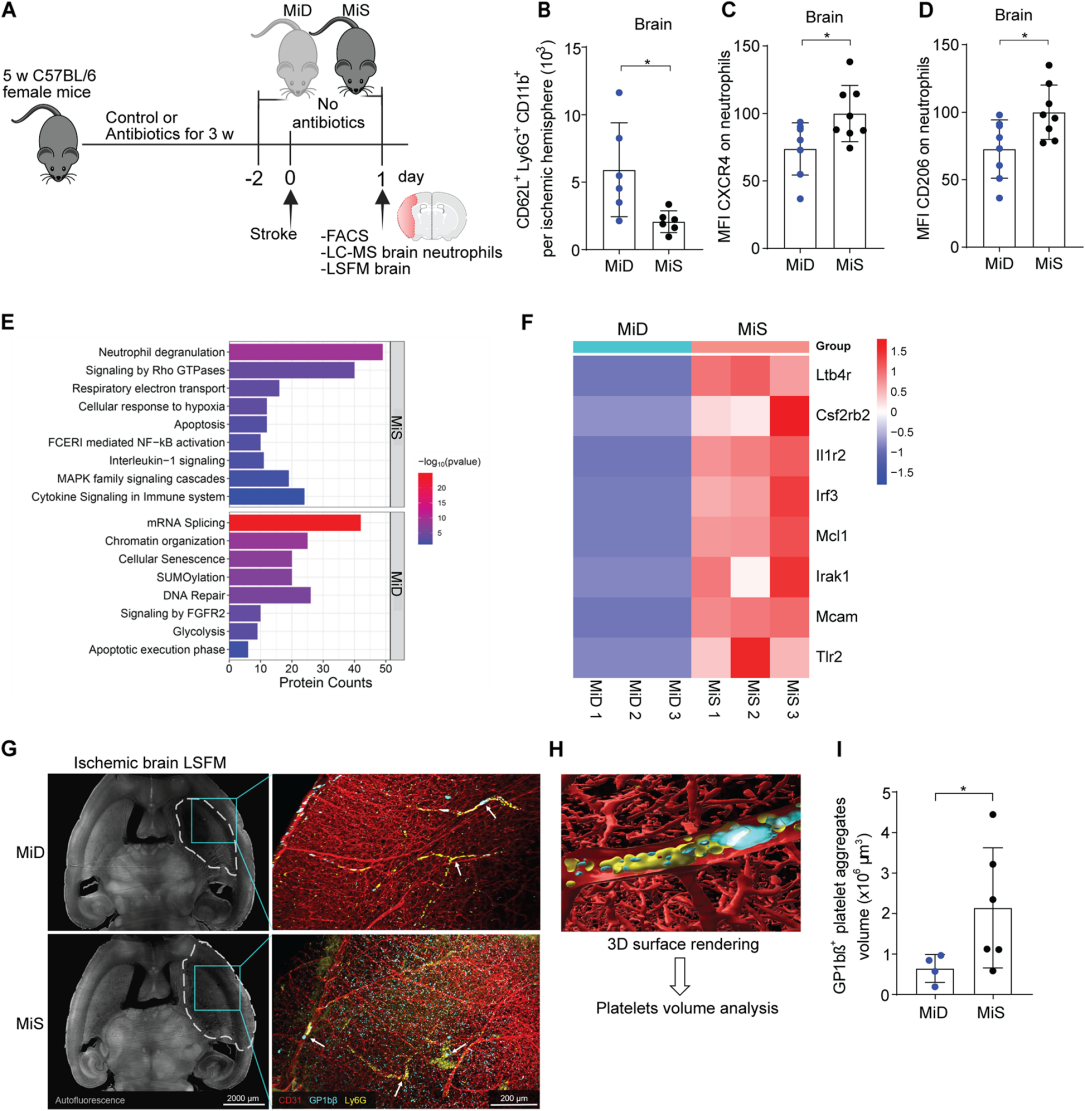
Gut microbiota support the appearance of activated neutrophils in the ischemic brain. **A** Schematic representation of the experimental paradigm. **B** Number of CD62L^+^Ly6G^+^CD11b^+^ neutrophils in the ischemic hemispheres of MiD and MiS stroke mice one day after surgery. **C**-**D** Mean fluorescence intensity (MFI) of CXCR4 and CD206 on brain-infiltrating neutrophils in MiD and MiS stroke mice. Values are normalized to MiS controls and presented as percentages relative to the 100% MiS mean. **E** Functional annotation of differentially regulated proteins between two groups. The biological processes with *p*-value < 0.05 were ranked by their protein abundance and the top eight pathways are shown here. **F** Heat map illustrating the proteins uniquely detected in ischemic brain-infiltrated neutrophils in MiS stroke mice but not in MiD mice. The color bar represents the relative z-score, which indicates relative abundance. Blue cells represent no abundance, while red cells represent the presence of the proteins. **G** LSFM brain imaging of MiD and MiS mice one day after stroke. (Left) Whole brain overview image, rendered on 100 µm slicer. Delineation of the infarct region visible in tissue autofluorescence (circled by a dashed white line). Scale bar, 2000 µm. (Right) 3D-rendered LSFM images of GP1bβ^+^ (cyan) platelet aggregates, Ly6G^+^ neutrophils (yellow) and CD31^+^ (red) brain vasculature of mice one day after stroke. White arrows indicate platelet aggregates and neutrophils in the ischemic brain. Scale bar, 200 µm. **H** High-resolution image showing 3D surface rendering for GP1b β^+^, Ly6G^+^, and CD31^+^ signals in ischemic brain vasculature. GP1b β^+^ surfaces were used for quantification of platelet aggregate volumes. **I** Quantification of total GP1b β^+^ aggregate volumes (× 10^6^ μm^3^) within CD31^+^ microvasculature of 6 mm^3^ ipsilateral brain region of MiD and MiS stroke mice. Data are analyzed using the unpaired Mann–Whitney U test for two-group comparisons, *p < 0.05, *n* = 4–8 mice per group. MiD = microbiota-deficient, MiS = microbiota-sufficient

Next, we investigated whether gut microbiota depletion influences neutrophil infiltration into ischemia-injured brain hemispheres and their maturation states (Fig. 4A). Our results showed that gut microbiota depletion increased brain-infiltrating CD62L^+^ immature neutrophils without affecting overall neutrophil invasion and Ly6G expression (Fig. 4B and Figure S4 A, B). Notably, brain-infiltrating neutrophils expressed decreased levels of the maturation receptors CXCR4 and CD206 in MiD mice compared to MiS mice (Fig. 4C, D). However, a similar expression of Ly6G was detected in both mice groups (Figure S4 C).

In additional studies, we characterized the proteomic changes of brain-infiltrating neutrophils after microbiota depletion. Comparative LFQ proteome analysis between MiD and MiS mice yielded a total of 4,778 proteins identified from both groups with 1% FDR, and 714 proteins were found to be differentially regulated between two conditions with log_2_fold change ± 0.58 and statistical significance level *p*-value < 0.05. Gene Ontology (GO) analysis of several differentially regulated proteins revealed that neutrophils from MiS mice showed upregulation in pathways related to the ‘neutrophil degranulation’ and inflammatory signaling pathways (NF-kB, IL-1, MAPK cytokines) (Fig. 4E). Among the 379 upregulated proteins in MiS mice, inflammatory proteins such as MMP8, MMP9, Chitinase-3-like protein 1, S100-A11, and Formyl peptide receptor 2, which are typically released during neutrophil degranulation, were abundant. In addition, we identified a distinct set of proteins that were uniquely detected in all replicates of the MiS group but were absent in the MiD group (Fig. 4F). This protein subset includes anti-apoptotic factors such as Mcl-1, which prevents programmed cell death, and TLR-2, a key receptor involved in neutrophil activation and the induction of proinflammatory cytokines [27]. Our results also showed increased levels of neutrophil chemotaxis-inducer leukotriene B4 receptor and IRF3, a transcription factor that promotes neutrophil recruitment [28] and cytokine production, respectively. Furthermore, the proinflammatory mediators IRAK1 and IL1R2, which drive inflammation via NF-κB and MAPK activation, were also enriched in the MiS group. These findings suggest that gut microbiota may increase neutrophil inflammatory responses after stroke and exacerbate disease progression.

Based on our findings that gut microbiota can induce systemic NET release and facilitate platelet aggregation in organ microvasculature [8], we hypothesized that microbiota-mediated neutrophil activation could also trigger vascular occlusion in the ischemic brain. To this end, we analyzed platelet aggregation in the cerebral vasculature of MiS and MiD stroke mice using three-dimensional light-sheet fluorescence microscopy (LSFM) (Fig. 4G). Our imaging data identified colocalization of neutrophils with GP1bβ^+^ platelet aggregates in the ischemic brain microvasculature, suggesting their contribution to reperfusion failure. We observed accelerated vascular damage and immune cell infiltration in the ischemic brain territories in MiS compared to MiD stroke mice. Furthermore, we quantified platelet aggregates in ischemic brain tissue using surface rendering-based volume analysis (Fig. 4H). Interestingly, we found significantly reduced total GP1bβ^+^ platelet aggregate volumes within the ischemic brain vasculature of MiD compared to MiS stroke mice (Fig. 4I).

## Discussion

Our work shows that gut microbiota elicits neutrophil activation after stroke, resulting in a significant increase in cerebral infarct size. These findings are consistent with previous studies reporting an association between systemic neutrophil activation and worse stroke outcome [1, 14]. Our results from two established models of microbiota research (germ-free mouse colonization and antibiotic-mediated microbiota depletion) provided evidence for higher neutrophil inflammatory responses under the influence of gut microbiota after stroke. It is of high clinical interest that gut microbiota changes after stroke impact inflammatory brain injury [4]. Using a microbiota transfer mouse model, previous studies have demonstrated the effects of altered gut microbiota from pancreatitis patients on neutrophil activation and systemic inflammation [29]. The gut microbiota colonization of germ-free (GF) mice in our study reveals that it triggers strong neutrophil activation and increases sensorimotor deficits and brain infarct volumes after three days of stroke. Neutrophils in microbiota-colonized GF mice expressed higher levels of maturation markers Ly6G [30], LFA-1 [31], and CXCR4 [32] compared to GF stroke mice. These activated neutrophils after stroke might contribute to increased neuroinflammation and deteriorated stroke outcome. On the contrary, we have previously shown that the colonization of GF mice induces neuroprotective responses in a mouse model of permanent (p) MCAO [33]. Two major differences between the mouse models can explain these discrepancies. First, transient MCAO (tMCAO) in mice induces larger brain infarcts and changes in gut microbiota that are absent in permanent MCAO (pMCAO) with smaller brain lesions [4]. Second, tMCAO induces substantial systemic immune changes compared to pMCAO with more confined neuroinflammatory responses [34].

Our results shed further light on the possibility of targeting gut microbiota as a therapeutic approach to control deleterious neutrophil responses after stroke. Depleting gut microbiota with antibiotics induced an immature neutrophil phenotype and decreased inflammatory brain injury. Similar to GF mice, neutrophils in microbiota-depleted stroke mice expressed lower levels of Ly6G and CXCR4, indicating a juvenile phenotype. Moreover, microbiota deficiency increased the percentage of CD62L^+^ juvenile neutrophils in the circulation of unoperated mice. However, some variations between the expression levels of maturation receptors between colonized GF (Ex-GF) and MiS mice were detected, which may arise due to later (in GF) and immediate (in SPF) gut colonization of mice after birth [18, 35]. These data are consistent with previous reports that antibiotic treatment of mice before stroke induction is neuroprotective by unknown mechanisms [13]. In this regard, our transcriptomics data showed reduced inflammatory gene expression in ischemia-injured brain hemispheres of mice with a deficiency of gut microbiota three days after stroke. Previous work indicates that gut microbiota depletion with antibiotics reduces neutrophil responses to systemic infection in neonatal mice [18]. Similarly, Zhang et al. have shown that gut microbiota is indispensable for neutrophil aging and triggers the generation of disease-promoting neutrophil subsets [36]. Our results uncover further associations between gut microbiota and neutrophil activation under ischemic stroke conditions. In particular, our LC–MS-based proteomics data revealed that circulating neutrophils from microbiota-depleted mice exhibit very low levels of inflammatory proteins and release reduced levels of NETs compared to gut microbiota-sufficient mice. Interestingly, brain-infiltrated neutrophils in microbiota-deficient stroke mice displayed reduced inflammatory proteome, suggesting a strong interplay between gut microbiota and neutrophils. Given that gut microbiota can train innate immunity by priming through PAMPs such as peptidoglycan, LPS [37], and beta-glucans, which has recently been shown to contribute to neutrophil activation [38, 39] or via the release of fermentation products such as short-chain fatty acids [40], we speculate that adverse stroke reaction in microbiota-sufficient mice may result from these biomolecular interactions. Thus, future studies should investigate how gut microbiota-derived components interact to influence neutrophil activation after stroke.

Multiple studies have indicated that activated neutrophils can induce cerebral tissue injury by releasing pro-inflammatory cytokines, free radicals, or NETs decorated with toxic enzymes [3, 41]. Conversely, neutrophils with specific phenotypes may also promote tissue regeneration [42–44]. With our identification of key upregulated extracellular proteins in circulating neutrophils from gut microbiota-depleted stroke mice, neutrophils with neuroprotective properties may exist. When we depleted neutrophils by anti-Ly6G antibody treatment in MiD mice, the infarct volumes were substantially increased compared to neutrophil-sufficient MiD mice, confirming that neutrophil activation under the influence of gut microbiota is detrimental to ischemia-injured brains and that neutrophils in the condition of gut microbiota deficiency might have neuroprotective functions [45, 46]. We suggest that reduction in neutrophil activation in MiD mice underlies the observed brain-protective effects after stroke. However, direct brain-repairing effects of some immature neutrophil subsets cannot be completely excluded and require further investigation [45, 46]. In addition, there is the possibility of an indirect effect of juvenile neutrophils in down-regulating pro-inflammatory responses of microglia/macrophages and lymphocytes. For example, we found reduced microglia activation in the absence of neutrophils in MiS mice but an increase in their activation in neutrophil-depleted MiD mice after stroke. Increased levels of NETs have been identified in stroke patients, which are associated with immunodeficiency [8] and worse clinical presentation [7]. Previous studies have also demonstrated the direct toxic effects of NETs on the survival of cardiomyocytes [47]. NETs can stabilize platelet aggregation and promote vascular thrombus formation in the injured brain after stroke [7]. In addition, activated neutrophils have been shown to obstruct cerebral capillaries and impair blood flow after stroke [2]. Consistent with these findings, we found neutrophils within platelet aggregates in the microvasculature of ischemic brain hemispheres. The fact that gut microbiota depletion significantly reduced platelet aggregates in the cerebral vasculature after stroke suggests its role in neutrophil activation and brain vascular occlusion.

One limitation of our experimental study was the inclusion of only young female animals, which was required for co-housing GF experiments. We are aware of the developmental, biochemical, and stroke outcome differences between the sexes. Therefore, further investigations including young and old male mice deserve attention. However, there is evidence for the beneficial effects of microbiota depletion on the outcome of stroke in young male mice [13].

Altogether, gut microbiota depletion reduced neutrophil activation and improved recovery after transient ischemic stroke. The fact that neutrophil removal from microbiota-deficient stroke mice further increased the extent of brain injury suggests the beneficial effects of immature neutrophils by still unknown mechanisms. Our results raise the possibility that targeting microbiota using antibiotics may reduce the disease-aggravating effects of activated neutrophils in stroke patients, which should be tested in clinical trials.

## Supplementary Information

Supplementary Material 1: Figure S1. Gut microbiota transfer induces neutrophil activation in multiple lymphoid tissues after stroke. A. Schematic of multicolor flow cytometry analysis. B-D. Percentages of CD62L^+^ neutrophils in the blood, spleen and tibial bone marrow of GF and Ex-GF mice three days after stroke surgery. The difference between GF and Ex-GF mice is about 1% (*p*<0.05). E-G. Percentages of CXCR4^+^ neutrophils in blood, spleen and tibial bone marrow of GF and Ex-GF mice three days after stroke surgery. H-J. Total number of Ly6G^+^CD11b^+^ neutrophils in the blood, spleen and tibial bone marrow of GF and Ex-GF stroke mice. n=4-7 mice per group. Data were analyzed by the Mann-Whitney U test, *p<0.05, **p<0.01. GF=germ-free, Ex-GF=colonized germ-free, BM=bone marrow.

Supplementary Material 2: Figure S2. Gut microbiota depletion reduces neutrophil activation without affecting their numbers in stoke mice. A. Scheme illustrating experimental design. B. Mean fluorescence intensity (MFI) of CXCR2 and C. CXCR4 on neutrophils in MiD and MiS unoperated mice. Values are normalized to MiS controls and presented as percentages relative to the 100% MiS mean. D-F. Total number of Ly6G^+^CD11b^+^ neutrophils in the blood, spleen and tibial bone marrow of MiD and MiS mice three days after stroke. *n*=7-8 mice per group. G. Heat map illustrating differentially expressed genes associated with proinflammatory response in ischemic brain hemispheres in MiD and MiS mice three days after stroke. H. The pathway analyses on inflammation-related genes in KEGG or Reactome pathways in ischemic brain hemispheres of MiD and MiS stroke mice. The presented data with a false-discovery rate (FDR)< 0.05 and a log_2_-fold change above 0.3 were selected. Data were analyzed by the Mann-Whitney U test, *p<0.05, MiD= microbiota deficient, MiS=microbiota sufficient.

Supplementary Material 3: Figure S3. Neutrophils alter microglial activation phenotype after stroke. A. Illustration of microglia counting on Iba1 stained brain sections from stroke mice three days after surgery. B. Quantification of microglia in the ipsilateral core region of MiD and MiS neutrophil-sufficient and neutrophil-deficient mice C. in ipsilateral perilesional areas of MiD and MiS neutrophil-sufficient and neutrophil-deficient mice. D. Representative fluorescence confocal microscopy images of Iba-1 stained brain sections and cellular skeletonization analysis steps for characterizing brain microglia/macrophages. E. Illustration of microglia morphological analysis on Iba1 stained brain sections of stroke mice. F. Quantification of the average microglia endpoints in the ipsilateral perilesional areas of MiD and MiS neutrophil-sufficient and neutrophil-depleted mice. G. Quantification of the average process length in ipsilateral penumbra region of MiD and MiS neutrophil-sufficient and neutrophil-depleted mice. H. The amounts of plasma TNF-α in MiD and MiS stroke mice. n=6-8 mice per group. Data were analyzed using the Kruskall-Wallis test for multiple comparisons or the Mann-Whitney U test for two-group comparisons. *p<0.05, **p<0.01, ***p<0.001, ****p<0.0001, MiD= microbiota-deficient, MiS=microbiota-sufficient.

Supplementary Material 4: Figure S4. Gut microbiota depletion does not influence neutrophil numbers in ischemic brain hemispheres after stroke. A. Schematic depiction of the experimental paradigm. B. Total numbers of Ly6G^+^CD11b^+^ neutrophils in ischemic hemispheres of MiD and MiS stroke mice one day after surgery. C. Mean fluorescence intensity (MFI) of Ly6G on brain neutrophils. Values are normalized to MiS controls and presented as percentages relative to the 100% MiS mean. MiD=microbiota-deficient, MiS=microbiota-sufficient.

Supplementary Material 5.

## Data Availability

The mass spectrometry proteomics data have been deposited to the ProteomeXchange Consortium via the PRIDE partner repository with the dataset identifier PXD060229 (Username: reviewer_pxd060229@ebi.ac.uk Password: fAyCeV9PVSLe). The RNAseq data have been deposited to the array express repository with the dataset identifier E-MTAB-14982.
