# Supplementary figures and images for: Gut microbiota deficiency reduces neutrophil activation and is protective after ischemic stroke
Source: J Neuroinflammation. 2025 May 23;22:137. doi: 10.1186/s12974-025-03448-w (PMC12100894; doi:10.1186/s12974-025-03448-w)

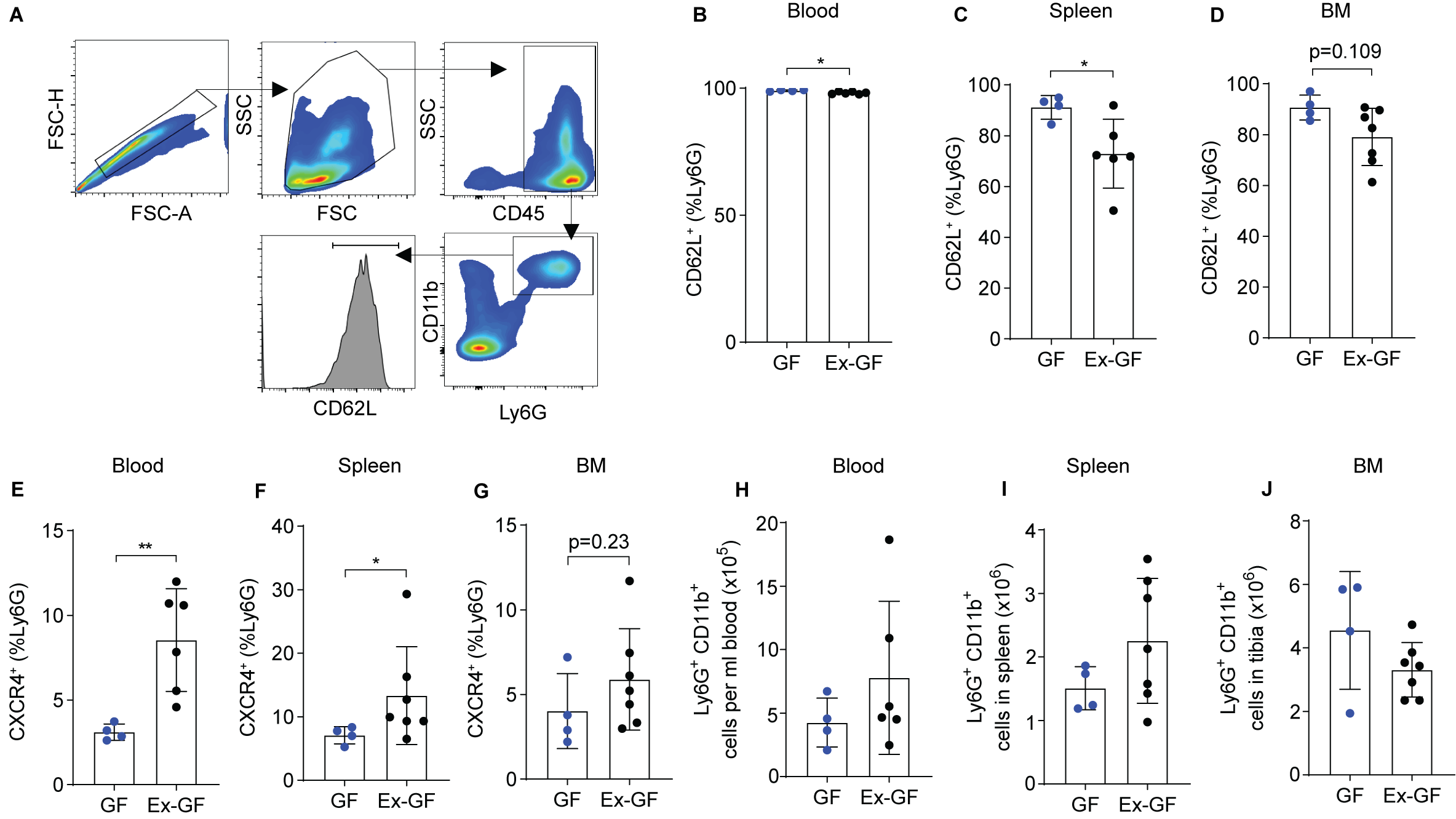

Supplement: Supplementary file 1 — Supplementary Material 1: Figure S1. Gut microbiota transfer induces neutrophil activation in multiple lymphoid tissues after stroke. A. Schematic of multicolor flow cytometry analysis. B-D. Percentages of CD62L+ neutrophils in the blood, spleen and tibial bone marrow of GF and Ex-GF mice three days after stroke surgery. The difference between GF and Ex-GF mice is about 1% (p<0.05). E-G. Percentages of CXCR4+ neutrophils in blood, spleen and tibial bone marrow of GF and Ex-GF mice three days after stroke surgery. H-J. Total number of Ly6G+CD11b+ neutrophils in the blood, spleen and tibial bone marrow of GF and Ex-GF stroke mice. n=4-7 mice per group. Data were analyzed by the Mann-Whitney U test, *p<0.05, **p<0.01. GF=germ-free, Ex-GF=colonized germ-free, BM=bone marrow. [file 12974_2025_3448_MOESM1_ESM.tif]

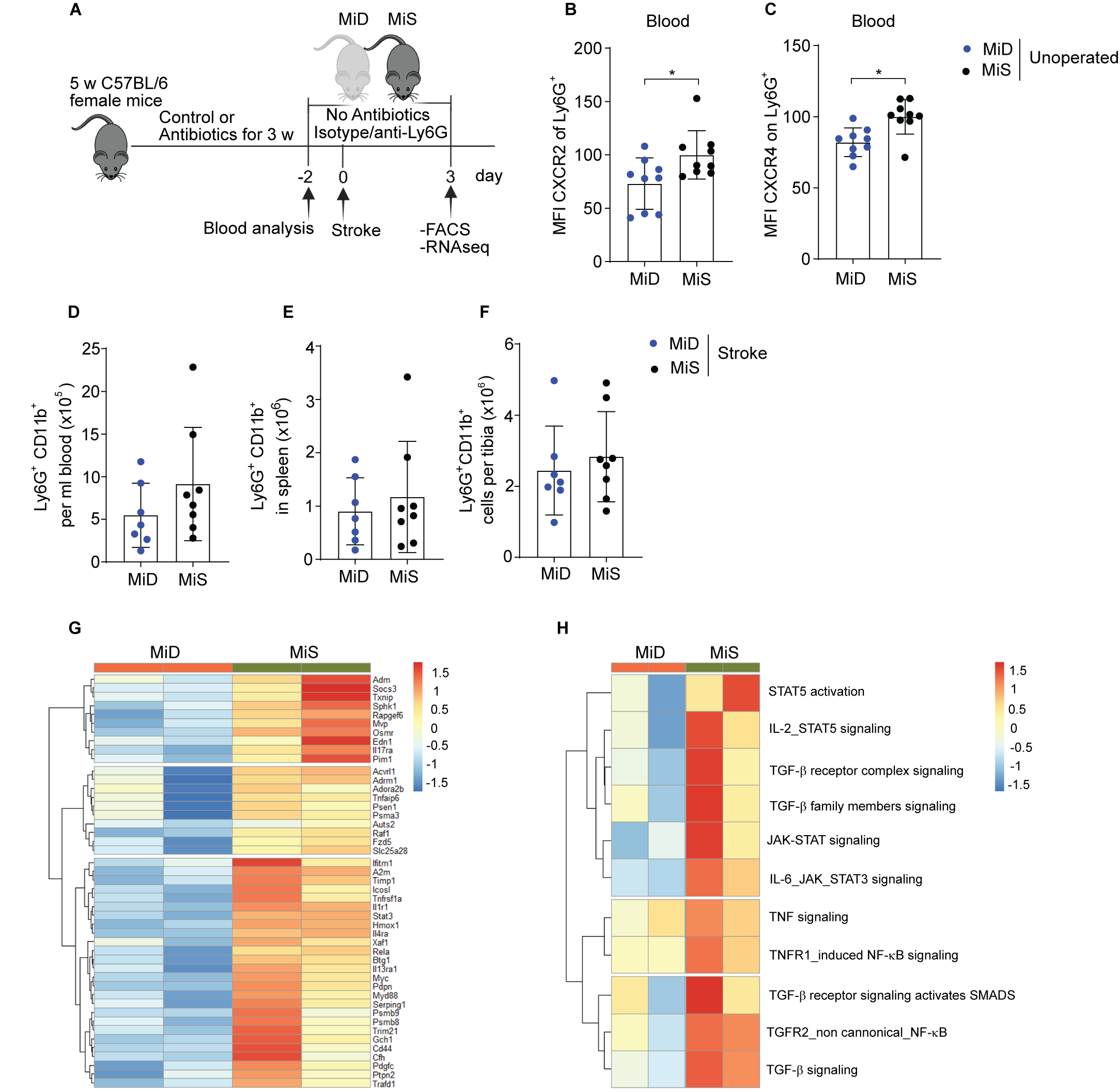

Supplement: Supplementary file 2 — Supplementary Material 2: Figure S2. Gut microbiota depletion reduces neutrophil activation without affecting their numbers in stoke mice. A. Scheme illustrating experimental design. B. Mean fluorescence intensity (MFI) of CXCR2 and C. CXCR4 on neutrophils in MiD and MiS unoperated mice. Values are normalized to MiS controls and presented as percentages relative to the 100% MiS mean. D-F. Total number of Ly6G+CD11b+ neutrophils in the blood, spleen and tibial bone marrow of MiD and MiS mice three days after stroke. n=7-8 mice per group. G. Heat map illustrating differentially expressed genes associated with proinflammatory response in ischemic brain hemispheres in MiD and MiS mice three days after stroke. H. The pathway analyses on inflammation-related genes in KEGG or Reactome pathways in ischemic brain hemispheres of MiD and MiS stroke mice. The presented data with a false-discovery rate (FDR)< 0.05 and a log2-fold change above 0.3 were selected. Data were analyzed by the Mann-Whitney U test, *p<0.05, MiD= microbiota deficient, MiS=microbiota sufficient. [file 12974_2025_3448_MOESM2_ESM.tif]

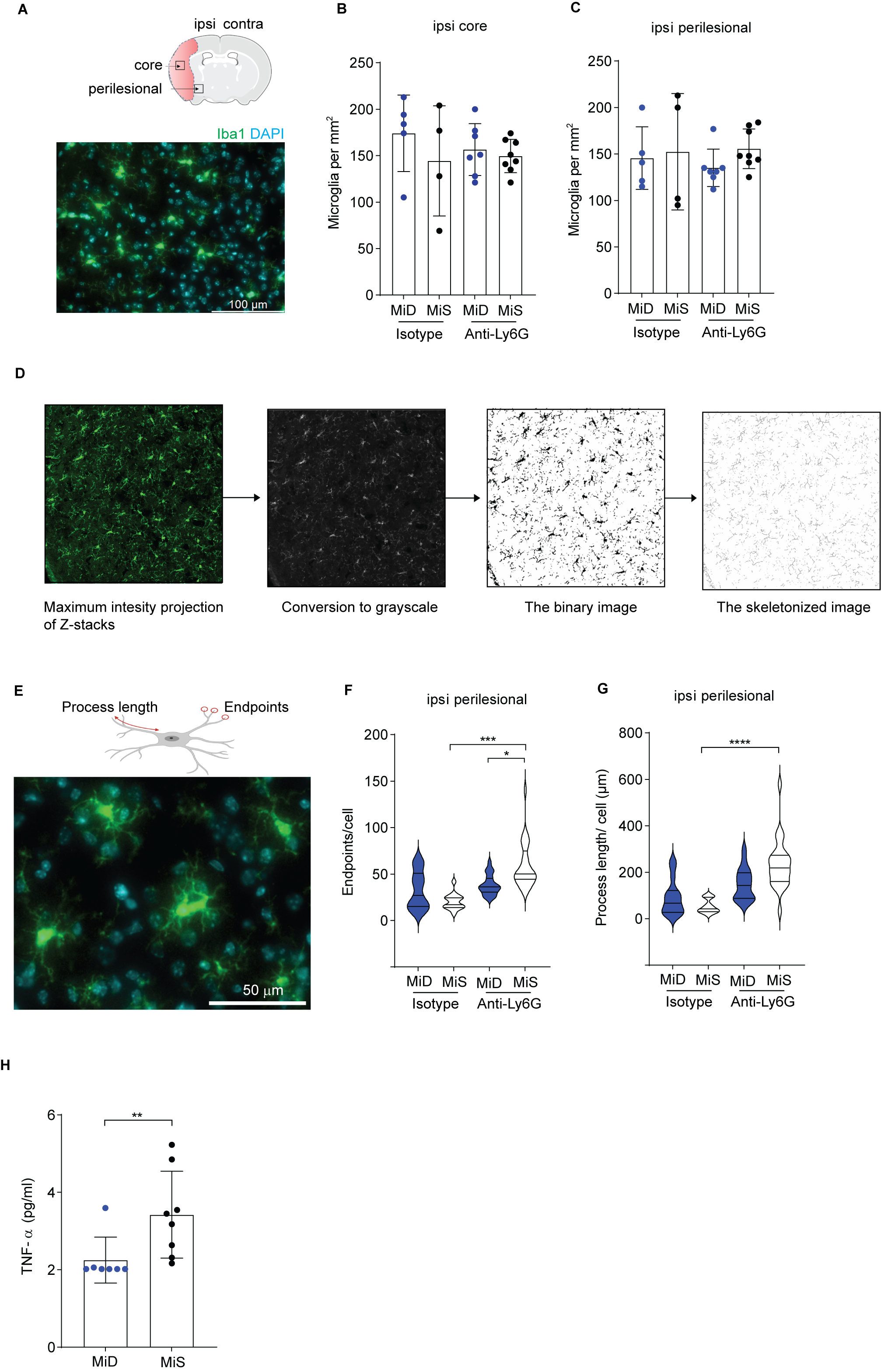

Supplement: Supplementary file 3 — Supplementary Material 3: Figure S3. Neutrophils alter microglial activation phenotype after stroke. A. Illustration of microglia counting on Iba1 stained brain sections from stroke mice three days after surgery. B. Quantification of microglia in the ipsilateral core region of MiD and MiS neutrophil-sufficient and neutrophil-deficient mice C. in ipsilateral perilesional areas of MiD and MiS neutrophil-sufficient and neutrophil-deficient mice. D. Representative fluorescence confocal microscopy images of Iba-1 stained brain sections and cellular skeletonization analysis steps for characterizing brain microglia/macrophages. E. Illustration of microglia morphological analysis on Iba1 stained brain sections of stroke mice. F. Quantification of the average microglia endpoints in the ipsilateral perilesional areas of MiD and MiS neutrophil-sufficient and neutrophil-depleted mice. G. Quantification of the average process length in ipsilateral penumbra region of MiD and MiS neutrophil-sufficient and neutrophil-depleted mice. H. The amounts of plasma TNF-α in MiD and MiS stroke mice. n=6-8 mice per group. Data were analyzed using the Kruskall-Wallis test for multiple comparisons or the Mann-Whitney U test for two-group comparisons. *p<0.05, **p<0.01, ***p<0.001, ****p<0.0001, MiD= microbiota-deficient, MiS=microbiota-sufficient. [file 12974_2025_3448_MOESM3_ESM.tif]

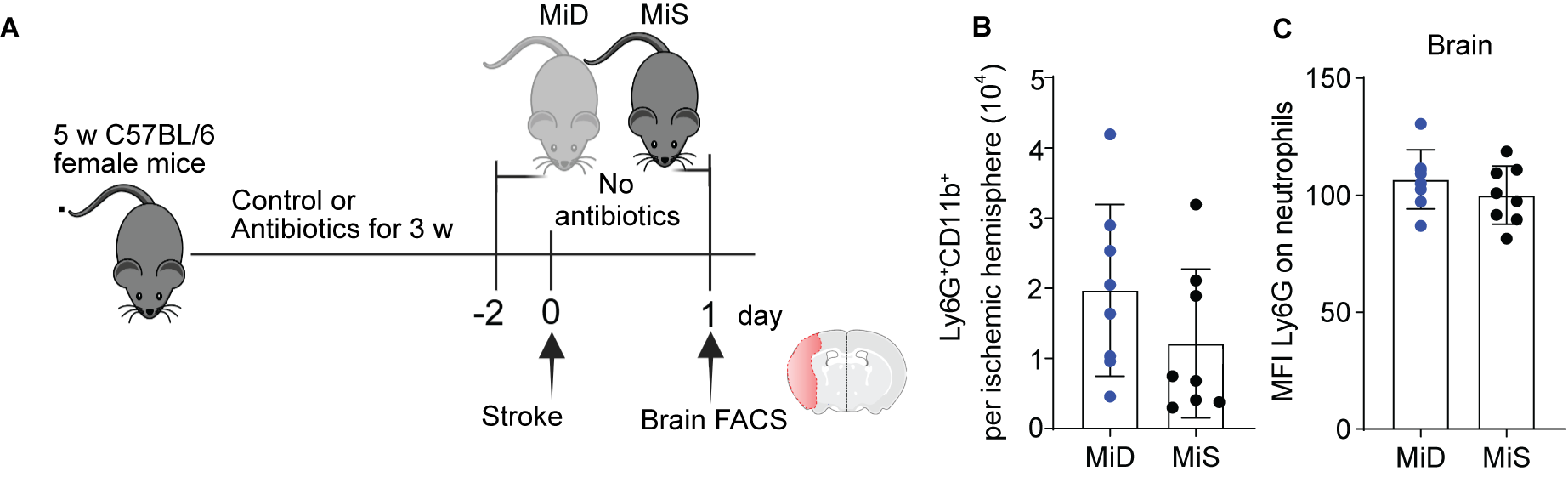

Supplement: Supplementary file 4 — Supplementary Material 4: Figure S4. Gut microbiota depletion does not influence neutrophil numbers in ischemic brain hemispheres after stroke. A. Schematic depiction of the experimental paradigm. B. Total numbers of Ly6G+CD11b+ neutrophils in ischemic hemispheres of MiD and MiS stroke mice one day after surgery. C. Mean fluorescence intensity (MFI) of Ly6G on brain neutrophils. Values are normalized to MiS controls and presented as percentages relative to the 100% MiS mean. MiD=microbiota-deficient, MiS=microbiota-sufficient. [file 12974_2025_3448_MOESM4_ESM.tif]
