# Supplementary material for: Gut microbiota deficiency reduces neutrophil activation and is protective after ischemic stroke
Source: J Neuroinflammation. 2025 May 23;22:137. doi: 10.1186/s12974-025-03448-w (PMC12100894; doi:10.1186/s12974-025-03448-w)
Supplement: Supplementary file 5 — Supplementary Material 5. [file 12974_2025_3448_MOESM5_ESM.docx]

**Methods**

**Mice experiments**

All animal experiments were performed consistent with guidelines for the use of experimental animals and were approved by the Regional Office for Nature, Environment and Consumer Protection of North Rhine-Westphalia, Germany (Landesamt für Natur, Umweltclarrr und Verbraucherschutz Nordrhein-Westfalen). Wild-type C57BL/6OlaHsd specific pathogen-free (SPF) female mice were obtained from Envigo, Netherlands. Germ-free (GF) C57BL/6OlaHsd female mice were obtained from the central germ-free animal facility of Hannover Medical School, Hannover, Germany. The SPF mice were housed in individually ventilated cages and had free access to food and water. GF mice were housed in sterile HAN-gnoto cages and received sterile water and food. All experiments were performed and reported according to ARRIVE guidelines^1^.

**GF mice and gut microbiota recolonization**

GF female mice were housed in sterile HAN-gnoto cages in the animal room dedicated only to sterile mice and a group of littermates (5 weeks old) were colonized by co-housing with SPF mice for six weeks. GF, Ex-GF (recolonized), or SPF mice received similar sterile food and drinking water throughout all experiments. Handling, behavioral testing, and surgical procedures with GF mice were performed under a UV-sterilized microbiological safety cabinet. The outer surface of the cage was carefully cleaned with Virkon-S solution (0.7 g/100 ml water) before opening it in the sterile safety cabinet. The surgeon wore a sterile surgical coat, mask, head cap, and gloves. For performing surgery in GF mice, all equipment used were autoclaved and/or UV sterilized. The sterility of GF mice after sacrifice was confirmed by plating fecal supernatants on blood agar plates. Similar procedures in the separate animal room were adapted for handling, behavioral testing, and surgery of Ex-GF mice.

**Depletion of intestinal microbiota**

The depletion of gut microbiota in SPF mice (5 weeks old) was achieved by administering four broad-spectrum antibiotics. The antibiotics employed in this study included ampicillin (1 g/L), metronidazole (1 g/L), neomycin sulfate (1 g/L), and vancomycin (0.5 g/L). These antibiotics were incorporated into the sterile drinking water of the mice for three weeks. To mitigate the bitter taste of the antibiotics, 5% sucrose was added to the drinking water. Mice in the control group received only 5% sucrose water for the same duration as the antibiotic-treated group. Two days before the stroke operation until sacrifice, the antibiotics and sucrose solutions were replaced with normal drinking water. The gut microbiota depletion was verified by the quantification of total fecal DNA and an increased cecum-to-body weight ratio.

**Microbial fecal DNA isolation and quantification**

Fecal samples were collected from GF mice after 6 weeks of co-housing with SPF mice to confirm successful recolonization. In experiments with antibiotics-mediated microbiota depletion, feces were collected after three weeks of treatment and three days after surgery. Fecal DNA was isolated using PowerFecal Pro DNA Kit as described by the manufacturer (Cat. 51804; Qiagen). The DNA concentration in samples was measured using Qubit 1x dsDNA HS assay-kit (Cat. Q33230; Thermo Scientific). Data was presented as total DNA per mg of fecal samples.

**Mouse model of ischemic stroke**

To induce ischemic stroke, a transient middle cerebral occlusion (tMCAO) was performed as described previously (Singh et al., 2021 BBI; Tuz et al. 2024 NCR). First, mice were anesthetized with 1% isoflurane in 100% oxygen and were injected with the analgesic buprenorphine (0.1 mg/kg, s.c.). A laser Doppler flow probe was affixed to the skull above the core of the middle cerebral artery (MCA) region. Mice were placed in a supine position on a feedback-controlled heating pad to maintain a body temperature of 37°C. An incision was made to expose the midline neck region and the common carotid artery (CCA) and left external carotid artery (ECA) were identified and ligated. A 2 mm silicon-coated filament (Cat. 702234PK5Re; Doccol) was inserted into the internal carotid artery (ICA) to occlude the MCA. Occlusion was confirmed by the corresponding decrease in Doppler flow signal to ≤20% of baseline. After 60 min of MCA occlusion, the filament was removed to reestablish blood flow and incisions were sutured. Mice were immediately injected with the anti-inflammatory drug carprofen (4-5 mg/kg, s.c.). For a postoperative period, mice were maintained in individual cages with ad libitum access to food and water. All animals were injected daily with carprofen (4-5 mg/kg, s.c.) until sacrificed. Mice were sacrificed after one or three days of ischemia-reperfusion injury. The severity of the stroke-induced cerebral injury was assessed using Clark’s point-based neurological scores (general+focal) **(Table 1, 2),** and behavioral deficits associated with a high neurological score were proportional to the worse stroke outcome.

**Antibody-mediated neutrophils depletion**

Neutrophils were depleted by the injection of anti-Ly6G antibody (Cat. BE0075-25, Bioxcell, 100 µg per mouse, i.v.) followed by anti-rat antibody (Cat. BE0122, 100 µg per mouse, i.p.) injection on the next day^2^. Mice underwent tMCAO and were injected with anti-Ly6G antibody (50 µg/mouse) and anti-rat antibody (50 µg per mouse, i.p.) the day following surgery. Mice received a final dose of anti-Ly6G antibody (50 µg/mouse) on the 2^nd^ day after surgery and were sacrificed three days after ischemia-reperfusion injury for analysis. This method efficiently depletes neutrophils expressing surface Ly6G, a surrogate marker for the identification of murine neutrophils. Depletion of circulating neutrophils was verified by intracellular staining with fluorochrome-conjugated Ly6G (1A/8) antibody using the FoxP3/transcription Factor staining kit (Cat. 00-5523-00, Thermo Scientific) followed by flow cytometry analysis.

**Flow cytometry**

Mice were deeply anesthetized with an injection of ketamin/xylazine (100 mg/kg and 10 mg/kg, i.p.) and blood was collected in EDTA tubes by cardiac puncture. Mice were then transcardially perfused with sterile PBS and spleen and bone marrow (BM) were isolated and placed in cold PBS. Plasma was isolated by centrifugation twice at 3000 g and 8000 g for 10 min at 4°C and stored at -80°C until further use. Single-cell suspensions were prepared by mincing spleens in PBS and filtering through a 70 µm cell sieve. Erythrocytes in blood, spleen, and tibial bone marrow samples were lysed using erythrocyte lysis buffer. Total cell counts were determined using an automated cell counter (Nexcelom Bioscience), and 500.000 cells per sample were used for antibody staining. The following anti-mouse antibodies were used: CD11b e450 (eBioscience), CD45 BV605 (BioLegend), CD62L PE (BioLegend), CXCR2 FITC (BioLegend), CXCR4 PerCP-Cyanine5.5 (BioLegend), CD206 PE-Cyanine7 (BioLegend), Ly6G APC (BioLegend). After a wash step in PBS, cells were resuspended in FACS buffer and measured on BD FACS Aria^TM^. Data were analyzed using FlowJo software (BD Biosciences). In our analysis, the mean fluorescence intensity (MFI) was defined from the positive population of the respective marker, calculating the geometric mean of each marker.

**Quantification of NETs in plasma samples**

The EDTA blood was centrifuged at 3000 g and the supernatant was collected for a second centrifugation at 8000 g for 10 minutes. The quantification of NETs was performed by a previously described capture ELISA, which is based on citrullinated histone H3 associated with DNA^3^. Briefly, antihistone H3 antibody (5 µg/ml; ab5103, Abcam) was coated overnight at 4°C onto 96-well plates followed by 5% BSA blocking for 2 h. The wells were then washed three times with 300 µl wash buffer followed by the addition of 50 µl plasma and 80 µl incubation buffer (including peroxidase-labeled anti-DNA antibody) for 2 h at 300 rpm (Cell Death ELISAPLUS, Cat. 11774425001, Roche). The wells were then washed three times with 300 µl washing buffer and 100 µl peroxidase substrate was added to the wells for 30 min in the dark. Then, 100 µl of ABTS peroxidase stop solution was added to the wells, and absorbance at 405 nm was measured and subtracted by absorbance at 490 nm (Abs 405 nm− Abs 490 nm). Absorbance values were considered in direct proportion to the amount of soluble NETs and were presented as a relative increase to control.

**Staining of brain vascular thrombi and optical tissue clearing**

Mice were deeply anesthetized with isoflurane in 100% oxygen 24 h after stroke surgery and i.v.-injected with fluorochrome-conjugated anti-CD31-APC/Fire750 (7.5 µg per mouse, BioLegend, Cat.102528, clone: MEC13.3), anti-GP1bβ-DyLight649 (3 µg per mouse, Emfret analytics, Cat. X-649) and anti-Ly6G-PE/Dazzle594 antibodies (7.5 µg per mouse, BioLegend, Cat.127648, clone: 1A8). Mice were then sacrificed by injection of ketamin/xylazine (100 mg/kg and 10 mg/kg, i.p.) and transcardially perfused with 15 ml PBS, followed by 15 ml 4% PFA (4 ml/min). Mice brains were removed and immediately fixed in 10 ml 4% PFA solution overnight at 4°C. The next day, brains were washed in PBS (4 h, 4°C) and transferred into a dehydration/delipidation series for optical tissue clearing. The samples were treated in serial dilutions of 15 ml tetrahydrofuran (THF, Roth, Cat.109-99-9) in PBS from 30% to 60% to 80% and twice 100% THF. Brains were incubated at each step for at least 8 h on an orbital shaker (75 rpm) at room temperature before being changed to the higher percentage solution. Finally, the brains were transferred to ethyl cinnamate (ECI, Sigma, Cat.112372) for at least 12 h before imaging to adjust the refractive index to achieve tissue transparency – a requirement for light sheet fluorescence microscopy (LSFM).

**Light sheet fluorescence microscopy for brain vascular thrombus analysis**

The stained and optically cleared brains were imaged by LSFM (UltraMicroscope BLAZE, Miltenyi Germany). The microscope systems use the ImSpector software (LaVision Biotec, Germany). The samples were positioned in the microscope chamber filled with 100% ECI using a steel holder.

For whole-organ overview images, brain autofluorescence was measured at 520/40 nm OPSL (Optically Pumped Semiconductor Laser; 50 mW) and detected with a 585/40 nm band-pass filter. Overview images were acquired using LVBT 1x objective, 0.6x zoom, and a step size of 10 µm, corresponding to a light-sheet thickness of 16.8 µm.

For detailed vascular analysis, two image stacks were acquired within the matching ischemic (ipsilateral) and non-ischemic (contralateral) hemisphere region, using the border of the external capsule for orientation to include lateral striatum portions as well as the cerebral cortex. Images were acquired in 6.64x magnification (objective LVBT 4x, zoom 1.66x) and a step size of 2 µm, adjusting the light-sheet thickness to 3.9 µm. Z-stacks covered a region of 1500 µm in the ventrodorsal direction, starting at bregma – 6.24mm and ending at bregma – 4.74mm, resulting in 750 images per hemisphere and measured channel.

Platelets stained with anti-GP1bβ-DyLight649 were excited/ measured on 630/30 nm OPSL and detected with a 680/30 nm bandpass filter. Endothelial cells stained with anti-CD31-APC/Fire750 were excited at 740/40 nm OPSL and detected at 824/55 nm. Neutrophils stained with anti-Ly6G-PE/Dazzle594 were excited with 560/40 nm and detected by a 630/30 nm band pass filter. For detailed images, the light-sheet width was adjusted between 30% and 50%. Brain 3D images were processed using Imaris software version 10.0.1 (Bitplane, Switzerland). The Imaris File Converter was used for image conversion. A magnification of 6.64x during imaging and a Z-range of 1500 µm per stack resulted in FOVs of 2000x2000x1500 µm, hence 6 mm^3^ of brain tissue analyzed per hemisphere. For data quantification, the Imaris surface function was applied separately upon the channels measuring the GP1bβ^+^ and Ly6G^+^ signals. To optimize the specificity of the rendered signals – indicating platelet aggregates or neutrophils – two subsequent filter settings were applied in the rendering process: First, signal-based, local threshold subtraction and second, volume-based exclusion of spheres below a volume of 3,3 µm^3^ for GP1bß^+^ and below 150 µm^3^ for Ly6G^+^. Inclusion of only GP1bß^+^ surfaces larger than 3,3 µm^3^ corresponding to the minimum mean murine single platelet volume of ~4,7 +/- 0,3 fl^4^ and subtraction of approximately 25% expected clearing shrinkage (for THF dehydration, 12h per step)^5^. Inclusion of only Ly6G^+^ surfaces above 150 µm^3^ referring to the minimum mean volume of a murine neutrophil (200-400 µm^3^)^6^ and 25% clearing shrinkage. Individually rendered GP1bβ^+^ surfaces indicate the number of thrombi within the imaged brain vasculature 24 h after stroke. Total GP1bß^+^ aggregate volumes [µm^3^] were quantified in the ischemic brain (micro-)vasculature of microbiota-sufficient (MiS) and microbiota-deficient (MiD) stroke mice.

**Quantification of brain lesion volumes**

For quantification of infarct volume, 20 µm sections at 500 µm intervals were cut from frozen brains on a cryostat. Cryosections were stained with cresyl violet solution (Cat. C5042, Sigma) and infarct volumes were calculated. Briefly, stained slides were scanned at 600 dpi and the open-source software ImageJ was used for analysis. The area of unstained infarct tissue and both hemispheres were measured at a scale of 23.62 pixels/mm. Swelling was calculated using the following formula: (ischemic area) = (direct lesion volume) − [(ipsilateral hemisphere) − (contralateral hemisphere)]. The total infarct volume (mm^3^ ) was calculated by integrating the measured areas and intervals between the sections.

**Purification of blood neutrophils for proteomics**

EDTA blood plasma was isolated and stored at −80°C. Blood samples were then incubated with 5 mL RBC lysis buffer (37°C) for 1 min to remove red blood cells. The reaction was stopped by adding 10 ml of PBS and the samples were centrifuged. After discarding the supernatant, the pellet was resuspended in 200 µl MACSQuant® Running Buffer and the neutrophils were sorted using the anti-Ly6G microbeads kit, mouse (Cat. 130-120-337, Miltenyi Biotec). Cells were washed twice in PBS to remove any buffer proteins. Cells were counted using an automated cell counter (Nexcelom Bioscience) and the cell pellet was stored at -80°C until further use.

**Brain single-cell suspensions for flow cytometry**

Mice were deeply anesthetized with an injection of ketamine/xylazine (100 mg/kg and 10 mg/kg, i.p.) and after transcardial PBS perfusion the brains were dissected and collected in sterile ice-cold HBSS, containing 15 mM HEPES buffer and 5% Glucose. The brain hemispheres were separated and the ipsilateral hemisphere was placed in a petri dish and chopped into small pieces using a scalpel. Afterwards, the petri dish was rinsed with 1 ml HBSS and the minced cerebral tissue was transferred into a falcon tube for centrifugation at 1500 rpm for 3 min. After discarding the supernatant, the brain tissue was resuspended in a 5 ml digestion mix, containing DMEM, supplemented with 100% FCS, 1% penicillin/streptamycin, 10 µg/ml DNase-I, and 5 µg/ml Liberase. The resuspended tissue was incubated for 10 min at 37°C with constant shaking. The digested tissue was mixed by up- and down pipetting through a 20-gauge needle attached to a syringe and passed through a 30 µm cell strainer. The strainers were rinsed with 5 ml DMEM, supplemented with 10% FCS and 1% Pen/strep. The cells were centrifuged for 6 min at 1500 rpm and resuspended in 40% Percoll. The suspension was placed on a 70% Percoll layer in a 15 ml tube and centrifuged for 30 min at 2100 rpm at RT. Finally, the cells were washed once with supplemented DMEM, followed by antibody staining for flow cytometry analysis.

**Purification of brain neutrophils for proteomics**

Brain single-cell suspensions were prepared as described above. Neutrophils from the brain cell suspensions were sorted using an anti-Ly6G microbeads kit (Cat. 130-120-337, Miltenyi). Afterward, neutrophils were washed once in PBS and snap-frozen on dry ice. Samples were stored at -80°C until further proteomic analysis.

**Proteomics analysis of neutrophils**

The cell pellet was lysed with 1% SDS lysis buffer followed by sonication for 10 min using Bioruptor. The total protein amount was estimated using the BCA assay. Approximately 4-5 µg protein obtained from the lysate was further reduced with 10mM dithiothreitol (DTT) and alkylated with 20mM iodoacetamide (IAA). Carbamidomethylated protein was further acidified with 5.5% phosphoric acid (H3PO4) and then digested using an S-trap micro column (ProtiFi), following the manufacturer’s protocol. Sequencing grade Trypsin was used to digest the protein at a 1:10 (enzyme: substrate) ratio for 2 h at 47°C. Peptides eluted from the column were vacuum-dried and reconstituted in 0.1% TFA. All peptides were spiked with iRT (Biognosys) peptides to maintain consistency and reproducibility across the runs. The cleaned peptides (~0.4µg) were analysed using timsTOF HT (Bruker Daltonics, Germany) coupled to an UltiMate® 3000 Nano LC system (Dionex). Peptides were separated on a 25 cm long and 75 μm I.D. C-18 reversed-phase Aurora column equipped with an integrated emitter (Ion Opticks) with a linear gradient of 3% to 35% solvent B (composed of 84% Acetonitrile and 0.1% formic acid) over 90 min was employed at a flow rate of 400 nl/min. Data were acquired in data-independent acquisition (DIA)- parallel accumulation serial fragmentation (PASEF) mode. The DIA-PASEF scheme was optimized in the py-diAID package based on our previous project-specific library. All ions were analyzed within the two-mobility window range covering from 0.6-1.6 1/K0 and 300-1200 m/z range with a cycle time of 2.1s.

The MS raw files were analyzed in Spectronaut v.18 (Biognosys) using the UniProt mouse database (UP000000589, downloaded on August 29, 2023) against the project-specific library. Other parameters were set as default BGS (Biognosys) settings. Identification of proteins, peptides, and precursors was achieved with 1% FDR. The area under the MS2 signals was considered for label-free quantification. SR plot were used for data visualization. Functional enrichment and gene ontology analysis were performed with the DAVID web server (DAVID knowledgebase v2023q4)29. The mass spectrometry proteomics data have been deposited to the ProteomeXchange Consortium via the PRIDE partner repository with the dataset identifier PXD060229 (Username: reviewer_pxd060229@ebi.ac.uk Password: fAyCeV9PVSLe).

**Immunohistological analysis of brain microglia**

PFA-fixed, frozen brains of stroke mice were used to collect 20 µm frozen cryosections at 500 µm intervals and stored at -80°C. For immunohistochemistry staining, slides were thawed and fixed with 4% PFA for 10 min at room temperature. After washing in PBS, brain sections were incubated in citrate buffer at 50°C for 20 min and washed again with PBS. Sections were then incubated with blocking buffer (PBS + 10% goat serum) for 30 min and then incubated with the primary antibodies rabbit anti-Iba1 antibody (Cat. 019-19741, Wako) at 4°C overnight. The following day, slides were washed in PBS and incubated with secondary antibodies goat anti-rabbit secondary antibody conjugated with Alexa Fluor 488 (Cat. A11008, Thermo Scientific;) for 2 h at room temperature. Sections were then incubated with nuclear stain DAPI (Lifetechnologies; 1:5000) for 5 min and washed in PBS. Slides were mounted with Fluoromount (Thermo Fisher Scientific). For further analysis, slides were imaged using Leica 6000 Microscope. The images were taken in different regions of the ipsilateral and the corresponding contralateral sides with 20x magnification by using Bin 2x2 (696 x 520) image format. The captured images showed overlaying channels of Iba1/DAPI for a diligent evaluation of the data. Cells in different regions are counted by using the Cell Counter plugin of Fiji (ImageJ). The number of the corresponding cells in every region was then divided by the calculated area (304574 µm^2^), obtaining the number of cells/µm^2^.

**Microglia structural analysis**

Microglial structural analysis was performed using a skeletonization pipeline on the acquired confocal images as previously described^7^. Z-stacks of 20 µm intervals were acquired at 25x magnification. Images were acquired using Leica SP8 MP and FLIM. The images were acquired with an image size of 1024 x 1024 pixels, an image depth of 8 bit and a pinhole of 1 µm. Microglial morphology was analyzed using the Fiji (ImageJ) AnalyzeSkeleton Plugin. Average endpoints and process length were quantified parameters to characterize microglia, obtaining information on the extent of branches/ ramifications – indicative of the microglial activation phenotype. First, Z-stack images were acquired and saved as maximum-intensity projections. To better visualize the positive staining, the image was converted to grayscale and the settings (brightness/contrast) were adjusted. After further increasing the contrast using Unsharp Mask and removing salt-and-pepper noise using Despeckle, the image was converted to a binary image using Threshold. To remove single-pixel background noise and gaps between processes, Despeckle, Close, and Remove Outliers were applied. The image was then skeletonized and analyzed using the AnalyzeSkeleton plugin.

**Brain RNA isolation and RNA sequencing analysis**

Total RNA was isolated from mouse brains that were carefully divided into ipsilateral and contralateral hemispheres and then the ischemic tissues were processed using commercially available RNA isolation kits (Invitrogen). The RNA quality and quantity were measured using nanodrop and Qubit assays (Thermo Fisher) and samples were stored at -80°C until further analysis. RNA sequencing libraries were prepared from 100 ng of total mouse brain RNA using the TruSeq Stranded mRNA (cat# 20020594, Illumina, San Diego, California, USA) after the depletion of ribosomal RNA. RNA libraries were sequenced on an Illumina Novaseq 6000 with 100 bp paired-end read (~60 million reads per sample). Fastq files were quality-checked with the Fastqc tool and the reads were trimmed with the Trimmomatic tool. Reads were mapped to the mouse genome (GRCm38 - mm10) and features were quantified using Htseq counts. Gene expression matrices with raw counts were processed with Deseq2 for differential gene expression and validated with edgeR and LIMMA^8^. For pathway analyses, single sample gene set enrichment analyses was performed using the gene set variation analysis Bioconductor package GSVA^8^. Inflammation-related hallmark, KEGG or Reactome pathways with a false-discovery rate (FDR) < 0.05 and a log_2_-fold change above 0.3 were selected and presented along with the enriched genes. The RNAseq data have been deposited to the array express repository with the dataset identifier E-MTAB-14982.

**Statistical analysis**

GraphPad Prism version 10.23 was used to analyze all data. Data is expressed as the mean ± s.d. Normality was tested using the Shapiro-Wilk normality test. For the comparison of non-Gaussian distributed groups, the Kruskal-Wallis test was used for more than two groups and two groups were compared via the two-tailed Mann–Whitney *U*-test. Statistically significant differences were defined as those with p-values ≤ 0.05.

**References**

1. Kilkenny, C., Browne, W., Cuthill, I.C., Emerson, M., Altman, D.G., and Group, N.C.R.R.G.W. (2010). Animal research: reporting in vivo experiments: the ARRIVE guidelines. Br J Pharmacol *160*, 1577-1579. 10.1111/j.1476-5381.2010.00872.x.

2. Boivin, G., Faget, J., Ancey, P.B., Gkasti, A., Mussard, J., Engblom, C., Pfirschke, C., Contat, C., Pascual, J., Vazquez, J., et al. (2020). Durable and controlled depletion of neutrophils in mice. Nat Commun *11*, 2762. 10.1038/s41467-020-16596-9.

3. Sun, S., Duan, Z., Wang, X., Chu, C., Yang, C., Chen, F., Wang, D., Wang, C., Li, Q., and Ding, W. (2021). Neutrophil extracellular traps impair intestinal barrier functions in sepsis by regulating TLR9-mediated endoplasmic reticulum stress pathway. Cell Death Dis *12*, 606. 10.1038/s41419-021-03896-1.

4. Jirouskova, M., Shet, A.S., and Johnson, G.J. (2007). A guide to murine platelet structure, function, assays, and genetic alterations. J Thromb Haemost *5*, 661-669. 10.1111/j.1538-7836.2007.02407.x.

5. Lugo-Hernandez, E., Squire, A., Hagemann, N., Brenzel, A., Sardari, M., Schlechter, J., Sanchez-Mendoza, E.H., Gunzer, M., Faissner, A., and Hermann, D.M. (2017). 3D visualization and quantification of microvessels in the whole ischemic mouse brain using solvent-based clearing and light sheet microscopy. J Cereb Blood Flow Metab *37*, 3355-3367. 10.1177/0271678X17698970.

6. Ghosh, S., Tuz, A.A., Stenzel, M., Singh, V., Richter, M., Soehnlein, O., Lange, E., Heyer, R., Cibir, Z., Beer, A., et al. (2024). Proteomic Characterization of 1000 Human and Murine Neutrophils Freshly Isolated From Blood and Sites of Sterile Inflammation. Mol Cell Proteomics *23*, 100858. 10.1016/j.mcpro.2024.100858.

7. Young, K., and Morrison, H. (2018). Quantifying Microglia Morphology from Photomicrographs of Immunohistochemistry Prepared Tissue Using ImageJ. J Vis Exp. 10.3791/57648.

8. Love, M.I., Huber, W., and Anders, S. (2014). Moderated estimation of fold change and dispersion for RNA-seq data with DESeq2. Genome Biol *15*, 550. 10.1186/s13059-014-0550-8.
